# Primary somatosensory cortex oscillations in trigeminal neuralgia: laser-evoked signatures and their potential relevance to microvascular decompression

**DOI:** 10.3389/fpain.2025.1652354

**Published:** 2025-09-29

**Authors:** Britta N. Kretzschmar, André Rupp, Sandro M. Krieg, Rezvan Ahmadi

**Affiliations:** ^1^ Department of Neurosurgery, University Hospital Heidelberg, Heidelberg, Germany; ^2^ Department of Neurology, University Hospital Heidelberg, Heidelberg, Germany; ^3^ Department of Neurosurgery, University Medicine Marburg - Campus Fulda, Fulda, Germany

**Keywords:** trigeminal neuralgia, MEG, magnetoencephalography, S1, primary somatosensory cortex, oscillations, microvascular decompression, laser-evoked fields

## Abstract

Classical trigeminal neuralgia (TN) is a severe chronic pain disorder characterized by sudden, intense facial pain attacks and represents a major burden for affected individuals. Microvascular decompression (MVD) can provide pain relief, yet not all patients benefit equally. A key challenge in selecting candidates for MVD lies in the limited predictive accuracy of current diagnostics, which mainly rely on subjective pain history and structural MRI findings. Since many asymptomatic individuals show neurovascular contact on imaging, its prognostic value remains limited. Electrophysiological measures, particularly cortical oscillations, may offer more objective insights into nociceptive system function. In this case series, we investigated 15 TN patients scheduled for MVD using magnetoencephalography prior to surgery to assess laser-evoked fields. Noxious stimuli were applied to the symptomatic and contralateral trigeminal dermatome. Ten patients achieved complete postoperative pain relief (responders), while five patients reported persistent symptoms (non-responders). Source reconstruction showed activation in the contralateral primary somatosensory cortex in all participants. Responders exhibited reduced low-frequency oscillatory activity at the pain site, whereas non-responders displayed increased activity in the same frequency band. Group-level analysis revealed distinct differences in oscillatory dynamics between responders and non-responders. These findings indicate altered cortical processing in TN and suggest that oscillatory activity patterns might serve as functional biomarkers. Incorporating these measures could improve preoperative stratification and guide treatment decisions for patients undergoing MVD.

## 1 Introduction

Trigeminal neuralgia (TN) is a severe facial pain disorder characterized by sudden, electric shock-like attacks within the trigeminal nerve distribution. These attacks can be triggered by innocuous stimuli such as touch, chewing, talking or drinking and may also occur spontaneously (1, 2). TN is typically categorized into three subtypes: (1) classical TN, associated with vascular compression of the trigeminal nerve; (2) secondary TN, resulting from structural pathologies such as cerebellopontine angle tumors, multiple sclerosis or arteriovenous malformations; and (3) idiopathic TN, in which no causative abnormalities are identified on diagnostic imaging (2, 3).

When pharmacological treatment is insufficient or causes intolerable side effects, surgical microvascular decompression (MVD) is recommended as the first-line surgical intervention for classical TN (4). While approximately 68.9% of patients experience immediate postoperative pain relief, a subset, about 2.6%, reports no benefit, even with continued medication (5).

The clinical diagnosis of classical TN is primarily based on patient history, whereas magnetic resonance imaging (MRI) is essential for excluding secondary causes (6). Advances such as 3T MRI and high-resolution sequences like CISS (constructive interference in steady state) have significantly improved visualization of cranial nerves and their cisternal course (7). MRI achieves high specificity and predictive value when a neurovascular contact is accompanied by structural nerve changes, such as atrophy, dislocation, indentation or flattening, at the trigeminal root entry zone (6). Most commonly, the superior cerebellar artery (SCA) is involved and associated with better outcomes after MVD (5). Despite these advancements, the diagnostic utility of structural MRI remains limited in clinical decision-making. MRI frequently reveals neurovascular contacts in asymptomatic individuals (8, 9), making it challenging to differentiate between benign anatomical variants and clinically relevant compressions. Many patients present with borderline imaging findings, and the presence of neurovascular contact alone does not necessarily predict pain severity or treatment outcome. As a result, clinicians are often confronted with diagnostic ambiguity, particularly in patients with conflicting clinical and radiological profiles. In some cases, individuals with clear neurovascular compression fail to benefit from surgery, while others with unremarkable MRI findings experience substantial postoperative relief. This discrepancy highlights a crucial limitation: structural imaging lacks the capacity to reflect the functional status of the trigeminal nociceptive system. Without objective functional biomarkers, treatment decisions rely heavily on subjective symptom reports and the clinician's judgment, which can lead to suboptimal patient selection for invasive procedures such as MVD. Thus, there is a pressing need for complementary diagnostic approaches that can provide insight into the neurophysiological underpinnings of TN and improve preoperative risk stratification.

The pathophysiology of TN is increasingly understood as multifactorial, shaped by the interaction of anatomical, genetic and neurophysiological factors (10). A genetic predisposition, especially involving variants in genes that govern membrane ion channel dynamics, may contribute to the emergence of a hyperexcitable neuronal phenotype (11).

Prolonged static or pulsatile vascular compression can result in structural damage to the trigeminal nerve, particularly demyelination, axonal loss and inflammation (12–14). This peripheral nerve injury may enable ephaptic crosstalk, wherein action potentials from non-nociceptive, demyelinated Aβ fibers aberrantly activate adjacent nociceptive Aδ and C fibers (12, 15). These peripheral alterations might induce central sensitization within the trigeminal nucleus or higher-order brain regions (16, 17). Sustained or aberrant neuronal activity within trigeminal circuits can enhance excitability of central neurons, contributing to central sensitization. In addition, axonal degeneration may lead to increased responsiveness of second-order neurons through mechanisms of deafferentation-induced hyperexcitability (10). Structural and functional neuroimaging studies in patients with TN have further demonstrated abnormalities in regions associated with pain modulation and affective processing (18), including reductions in grey matter volume and disrupted connectivity within networks (19, 20).

During acute pain, a widespread network of brain regions is consistently activated, including the primary (S1) and secondary (S2) somatosensory cortices, the insula, the anterior cingulate cortex (ACC), the prefrontal cortex and the thalamus (21, 22). While experimental pain paradigms, typically involving brief, evoked noxious stimuli, reliably activate a well-defined network of brain regions, the cortical representation of chronic neuropathic pain —defined as *pain arising as a direct consequence of a lesion or disease affecting the somatosensory system* (23)— often appears less robust and more variable. It is also important to note that evoked responses may only partially capture the complexity of clinical pain, which often includes spontaneous and affective components.

Unlike the robust and reproducible activation patterns seen in experimental pain paradigms, chronic neuropathic pain is associated with reduced cerebral blood flow in the thalamus and other pain-related brain regions (24). Long-term neuroplastic alterations in chronic neuropathic pain are observed at multiple levels of the nervous system, including the spinal cord and cortical structures (25). At the neuronal level, mechanisms such as lowered activation thresholds, ectopic spike generation, altered receptor sensitivity, disinhibition and aberrant connectivity patterns have been identified (26). Altered neural plasticity in the context of neuropathic pain has been consistently demonstrated across multiple brain regions. Notably, cortical structures such as S1 and the ACC are frequently implicated in the persistence and processing of neuropathic pain (27, 28). Studies have highlighted that chronic pain conditions are not merely localized phenomena but involve widespread disturbances in large-scale brain networks. In particular, functional coupling between the salience network and other pain-related systems, including the ascending nociceptive pathway, descending modulatory circuits and the default mode network, has been shown to be disrupted across multiple frequency bands (theta, alpha, beta, gamma) (29). Furthermore, these cross-network abnormalities differ between neuropathic and non-neuropathic pain states and their spectral characteristics have been linked to clinical pain severity. Such broadband coupling alterations suggest that chronic pain may be characterized by aberrant oscillatory communication across distributed networks, providing a potential systems-level framework for understanding the complexity and variability of pain experiences.

In TN, several neuroimaging studies have reported alterations in brain structure, function and connectivity (30, 31–34). Functional alterations in large-scale networks involved in facial pain processing, such as the default mode, sensorimotor and salience networks, have been observed in patients with TN (30). Structural MRI studies show reduced gray matter volume in S1 and thalamus in TN patients compared to healthy controls (35). Furthermore, patients with TN and neurovascular contact exhibited widespread grey matter reductions in regions associated with bottom-up pain processing, such as the insula, somatosensory cortex and thalamus, whereas those without neurovascular contact showed more localized deficits, primarily in the prefrontal cortex, suggesting altered top-down modulation (36). Diffusion tensor imaging (DTI) has demonstrated compromised white matter integrity in pathways connecting the thalamus to various cortical regions, including the somatosensory cortex in individuals with classical TN (34, 37). Given these findings, cortical mechanisms seem to play a significant role in the pathophysiology of TN (38). Therefore, future diagnostic strategies should aim to capture functional changes not only in the peripheral trigeminal system but also in cortical and subcortical processing pathways.

Laser-evoked potentials (LEPs) are a reliable method for assessing small fiber function, as they selectively activate A*δ* and C fibers while bypassing large myelinated Aβ fibers (39). In the context of TN, Squintani et al. (40) demonstrated an association between neurovascular compression and Aδ fiber impairment, suggesting that LEPs might be more sensitive than conventional trigeminal reflexes in detecting small fiber dysfunction.

Laser stimuli represent discrete sensory events that induce transient changes in the ongoing electroencephalogram (EEG) or magnetoencephalogram (MEG), resulting in evoked potentials or fields (EP/EF) as well as event-related modulations in the amplitude or power of oscillatory brain activity. Neurophysiological investigations of evoked pain have traditionally focused on the time-domain characteristics of evoked responses (41). To improve the signal-to-noise ratio of these responses, across-trial averaging is commonly applied. However, this approach selectively captures phase-locked activity and might obscure induced (non-phase-locked) oscillatory components that carry important functional information (42).

Time-frequency decomposition techniques overcome this limitation by enabling the analysis of both evoked and induced oscillatory activity, thus providing a more comprehensive representation of the dynamic cortical responses to noxious stimuli (43, 44).

The aim of this study was to investigate induced cortical oscillations in patients with classical TN scheduled for MVD. By neurophysiologically characterizing responders and non-responders, we sought to identify specific oscillatory patterns associated with surgical outcomes. Such patterns might offer predictive value for surgical success and pave the way for more personalized diagnostic and therapeutic approaches in the future.

## 2 Materials and methods

### 2.1 Participants

We enrolled 18 patients with classical TN scheduled for MVD at the Department of Neurosurgery, Heidelberg University Hospital. Patient characteristics are summarized in Table 1. Three participants were excluded from further analysis due to excessive muscle artifacts or tremor during MEG recording.

**Table 1:** Clinical characteristics of patients with classic trigeminal neuralgia (TN) who underwent microvascular decompression (MVD).

Patient	Dermatome	Diagnoses	MRI	Medication	Duration	io finding	Responder
1	Right V3	Lumbar disc herniation, s/p TBE	SCA conflict	Carbamazepine, amitriptyline, gabapentin, novaminsulfone, oxacarbazepin	2 years	Arterial nerve impression	No
2	Right V2	Hypothyreodism	SCA conflict	Gabapentin, lamotrigine, duloxetine	3 years	Arterial and venous nerve impression	Yes
3	Right V3	–	SCA conflict	Carbamazepine, pregabalin	8 years	Arterial and venous nerve impression	Yes
4	Right V3	–	SCA conflict	Carbamazepine	2 years	Arterial nerve impression	No
5	Right V3	s/p MI, arterial hypertension	SCA conflict	Carbamazepine, novaminsulfone	12 years	Arterial nerve impression	Yes
6	Right V3	Arterial hypertension	SCA conflict	Carbamazepine, amitriptyline	<1 years	Arterial nerve impression	Yes
7	Right V3	–	Nerve atrophy	Carbamazepine, gabapentin	<1 years	Arterial nerve impression	No
8	Right V3	s/p left MVD	No typical conflict	Carbamazepine	5 years	Venous nerve contact	No
9	Left V3	Anxiety disorder, s/p ophthalmic zoster, neuroforaminal stenosis	SCA conflict	Morphine, carbamazepine	<1 years	Arterial nerve impression	Yes
10	Right V2	Atopic dermatitis	SCA conflict	Oxcarbazepine, topiramate	7 years	Arterial nerve impression	Yes
11	Left V3	Arterial hypertension	SCA conflict	Carbamazepine, oxycodone, gabapentin	1.5 years	Arterial and venous contact, no impression	No
12	Left V2	–	SCA conflict	Carbamazepine, pregabalin, baclofen	8 years	Arterial nerve impression	Yes
13	Right V3	Arterial hypertension	SCA conflict	Carbamazepine, amitriptyline, novaminsulfone	<1 years	Arterial nerve impression	Yes
14	Right V3	–	SCA conflict	Gabapentin, ibuprofen	1 years	Arterial and venous impression	Yes
15	Left V2	Arterial hypertension	No typical conflict	Carbamazepine	14 years	Arterial nerve impression	Yes

Intraoperative classification of neurovascular compression was based on visible arterial or venous impressions on the trigeminal nerve. Patients were defined as responders if they achieved complete pain relief within four weeks postoperatively. io, intraoperative; SCA, superior cerebellar artery; TBE, tick-borne encephalitis; MI, myocardial infarction; MRI, magnetic resonance imaging; MVD, microvascular decompression.

Pain attacks were strictly unilateral and localized either in the maxillary (V2) or mandibular (V3) branch of the trigeminal nerve. Eleven patients identified V3 as the primary affected dermatome, with eight patients experiencing pain in the right V3 and three patients in the left V3. In the group of patients with symptoms in the V2 dermatome, the pain attacks were localized in two patients on the left side and in two patients on the right side.

All patients continued their prescribed analgesic medication throughout the study period. Follow-up four weeks after surgery identified ten patients with complete pain remission, constituting the responder group. Five patients who reported persistent pain despite surgery and ongoing medication formed the non-responder group.

MEG data were recorded one day prior to surgery. The final sample included nine female and six male patients, aged between 35 and 70 years. Fourteen participants were right-handed, and one was left-handed.

All patients provided written informed consent before participating. The study protocol was approved by the Institutional Review Board of Heidelberg University (Study ID: S-815/2019). No patient reported somatosensory dysfunction outside the affected trigeminal dermatome. All participants underwent a neurological assessment by a board-certified neurosurgeon and presented with a clinical history consistent with classical TN.

The responder group (*n* = 10) consisted of patients who reported complete absence of trigeminal pain attacks and remained entirely pain-free four weeks after MVD. The non-responder group (*n* = 5), in contrast, was defined by persistent pain symptoms despite continued use of medication.

### 2.2 Stimulation paradigm and recording technique

Before MEG data acquisition, we determined individual laser intensity by applying noxious laser pulses (Nd:YAP, Stimul 1,340, El.En., Florence, Italy; wavelength: 1,340 nm, duration: 3 ms, diameter: 5 mm) of increasing intensity (0.5–2.0 J in 0.25 J increments) outside the recording room to three areas: the dorsum of the right hand, the affected trigeminal dermatome and the corresponding contralateral control site. The intensity was increased until patients reported the stimulus as no longer tolerable. The individual stimulation intensity used during MEG recordings was selected to be both tolerable and to elicit a pain rating of at least 50 on the NRS (numerical rating scale) ranging from 1 to 100. Seven patients tolerated a painful pricking sensation at 2.00 J (∼101.86 mJ/mm^2^), six patients at 1.75 J (∼89.13 mJ/mm^2^), and two patients reached their limit at 1.50 J (∼76.39 mJ/mm^2^).

The gradients of the magnetic fields were recorded using a 122-channel whole-head MEG system (Neuromag, Elekta Oy, Helsinki, Finland) in a magnetically shielded room (Imedco, Hägendorf, Switzerland). Patients were seated in a comfortable chair, wore laser safety goggles and were instructed to keep their eyes open and focus on a monitor for at least 10 s after each laser stimulus without moving.

Prior to the MEG recording, four head position indicator (HPI) coils were attached to the patient's scalp and digitized together with 100 additional surface points at anatomical landmarks (Polhemus 3D Space Isotrack II, Colchester, USA). At the beginning of each recording, the position of the HPI coils within the dewar was measured to allow for accurate head localization. The digitized head points were later used to fit a spherical head model for source reconstruction. MEG signals were sampled at 1,000 Hz and low-pass filtered at 330 Hz during acquisition.

During MEG recording, patients received 30 noxious laser pulses [interstimulus interval (ISI): ∼10 s] at the affected dermatome (TN site) and the contralateral control site. To precisely target the invisible laser beam, a He-Ne laser was used to guide placement on the skin. After each stimulus, the beam was repositioned to a different skin site to avoid tissue damage and minimize nociceptor fatigue or sensitization.

Preoperative MRI data were primarily derived from externally performed cranial MRI scans utilizing the CISS sequence. Radiological findings were extracted from the corresponding written reports. Intraoperatively, a neurosurgeon visually documented the presence of significant neurovascular contact or nerve compression.

### 2.3 Preprocessing of recorded data

To reduce contamination from ocular, head movement and muscle activity, we applied an artifact correction approach based on principal component analysis (PCA) to remove components corresponding to these sources (45). Correction was computed from a −500 to 3,000 ms event window, targeting low-frequency (1–7 Hz) activity typically associated with ocular and head movement artifacts, and high-frequency (40–240 Hz) activity related to muscle artifacts (46). The first one to three principal components, typically representing non-neural sources, were removed from the data. Cleaned single trials were subsequently segmented into epochs from 1,000 ms before to 9,000 ms after stimulus onset. On average, 20–30 artifact-free trials were retained per recording session and used for subsequent source analysis and time-frequency decomposition.

### 2.4 Source estimation and time-frequency analysis

We defined the baseline interval from −100 to 0 ms. To analyze oscillatory activity at the source level, we employed a locally fitted spheres approach (47) as implemented in the Brainstorm toolbox (48). This method fits one local sphere beneath each sensor to construct the forward model. For anatomical alignment, we used the Colin 27 template brain (49), scaled individually to match each patient's head shape.

Noise covariance was computed using a baseline window from −500 to 0 ms. To localize S1 activity on the cortical surface, we applied a weighted minimum norm estimate (wMNE) (50). The baseline was normalized using a *z*-score transformation based on the interval from −100 to 0 ms. After normalization, source reconstruction revealed pronounced cortical activation in contralateral S1 within a temporal window spanning 50 ms before to 50 ms after the first peak in the superimposed gradiometer waveforms. Dipoles with constrained orientation were seeded at the activity center. The region of interest was then expanded using a correlation criterion of *r* = 0.95, starting from the vertex with the maximum amplitude.

Source waveforms were analyzed by averaging epochs from −100 to 500 ms relative to stimulus onset. A band-pass filter from 0.5 Hz to 15.0 Hz was applied after removing linear trends and performing direct current (DC) correction (baseline: −100–0 ms). For source-level time-frequency analysis (TFA), we applied a Morlet wavelet transform using a sliding Hanning window to cover the 1–30 Hz frequency range (mother wavelet center frequency fc = 1 Hz; time resolution FWHMtc = 2 s). Power values were averaged across trials.

To examine induced, non-phase-locked oscillations, we subtracted the power of evoked components from the total power. The analysis was conducted in a time window from −500 ms to 1,000 ms relative to stimulus onset. We subsequently applied a 1/f correction and calculated *z*-scores of spectral activity relative to the baseline (–500–0 ms). Final results were visualized as power transformations derived from the time-frequency decomposition.

For descriptive statistics, patients were stratified into responders and non-responders based on postoperative outcomes four weeks after MVD. Between-group comparisons were conducted to evaluate neurophysiological differences related to clinical outcome. For analysis of EF, source-reconstructed N2 m components were evaluated using one-sample *t*-tests against zero for each group and stimulation site. Latency differences between sites and groups were assessed using non-parametric permutation tests (1,000 permutations). For TFA, spectral power was computed across epochs and compared to baseline (–500–0 ms) using Student's *t*-tests. Significant power modulations within each group were first identified, followed by inter-site (TN vs. control) and inter-group (responders vs. non-responders) comparisons using cluster-based permutation testing. A cluster-forming threshold of *p* < 0.05 was applied, and significance was defined as clusters exceeding the 95th percentile of the permutation distribution. Analyses were performed using the Brainstorm toolbox.

## 3 Results

Preoperative MRI findings revealed a neurovascular conflict involving the SCA in twelve patients. Two patients showed no radiologically typical neurovascular conflict, and one patient presented with trigeminal nerve atrophy.

Intraoperative assessment confirmed a significant arterial impression on the trigeminal nerve in 13 patients. Additionally, in four of these patients, a concomitant venous conflict was observed. One patient exhibited both arterial and venous contacts without visible signs of nerve indentation, and another patient displayed a venous contact only (see Table 1).

Ten patients experienced complete pain relief four weeks postoperatively and were categorized as responders. Five patients continued to report residual pain despite surgery and ongoing medication and were classified as non-responders. During MEG recordings, no patient reported neuralgic pain attacks in response to the contactless thermal laser stimulation.

**Figure 1:**
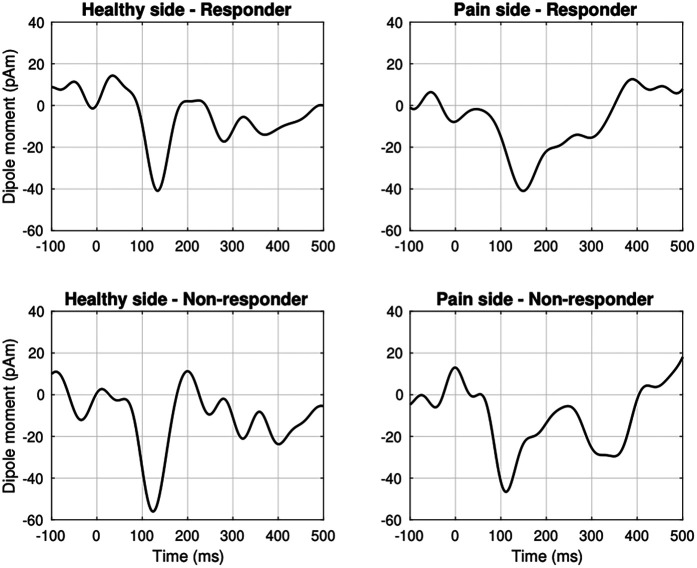
Grand-average source waveforms evoked by noxious laser stimulation in the responder group (*n* = 10) revealed a significant phasic N2 m peak in the contralateral primary somatosensory cortex (S1) at the control site [*t*-test against zero: 136 ms, *t*(9) = −4.5, *p* = 0.01] and the symptomatic dermatome [149 ms, *t*(9) = −4.5, *p* = 0.01]. In the non-responder group (*n* = 5), N2 m components were present with a latency of 125 ms at the control site [*t*(4) = −1.7, *p* = 0.07] and 115 ms at the affected side [*t*(4) = −2.5, *p* = 0.05]. Data were filtered using a 0.5–15.0 Hz band-pass filter.

Cortical source reconstruction consistently revealed activation in contralateral S1 across all patients (Supplementary Table S1). In the responder group (*n* = 10), grand-average source waveforms exhibited significant N2 m components at both the control site [*t*-test against zero: 136 ms, *t*(9) = –4.5, *p* = 0.01] and the TN site [149 ms, *t*(9) = –4.5, *p* = 0.01; Figure 1]. No significant latency difference was observed between the control and TN sites in the temporal domain [permutation test: 149 ms, *t*(9) = 1, *p* = 0.2].

In non-responders (*n* = 5), N2 m waveforms were observed at the control site with a latency of 125 ms [*t*(4) = –1.7, *p* = 0.07] and at the TN site with a latency of 115 ms [*t*(4) = –2.5, *p* = 0.05; Figure 1]. However, no significant difference emerged when comparing TN-site latencies between responders and non-responders [permutation test: 153 ms, *t*(13) = 0.3, *p* = 0.6].

**Figure 2:**
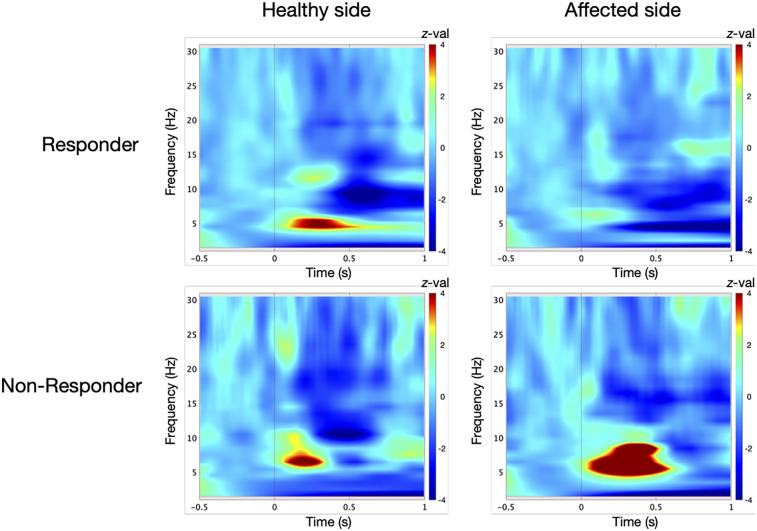
Induced low-frequency oscillatory responses in the contralateral primary somatosensory cortex (S1) were analyzed for ten responders and five non-responders at the control site and the affected trigeminal dermatome prior to microvascular decompression (MVD). Oscillatory power is displayed as *z*-scores relative to a 500 ms baseline. In responders, time-frequency analysis revealed a significant power increase at the control site [*t*-test against baseline: 5 Hz/242–325 ms; *t*(9) = 9, *p* = 0.01]. At the affected side, the oscillatory response was diminished compared to the control site [*t*-test against baseline: 6 Hz/8–188 ms and 5 Hz/25–28 ms; *t*(9) = 2, *p* = 0.05]. In contrast, non-responders exhibited a significant power increase at the affected dermatome [*t*-test against baseline: 7 Hz/280–352 ms; *t*(4) = 23, *p* = 0.01], which exceeded the response observed at the control site [*t*-test against baseline: 6 Hz/126–224 ms; *t*(4) = 9, *p* = 0.01].

**Figure 3:**
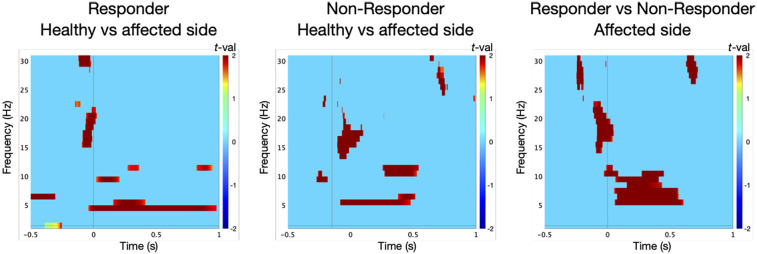
Statistical comparison of low-frequency oscillations in the contralateral primary somatosensory cortex (S1) in ten responders to microvascular decompression (MVD) revealed significantly lower oscillatory responses at the affected trigeminal dermatome (TN site) compared to the control site [permutation test: control site vs. TN site, 4 Hz/105–176 ms; *t*(9) = 3, *p* = 0.05]. In contrast, non-responders (*n* = 5) showed a significant increase in oscillatory power at the TN site relative to the control site [permutation test: TN site vs. control site, 5 Hz/31–114 ms; *t*(4) = 2, *p* = 0.05]. Moreover, non-responders exhibited distinct oscillatory dynamics at the affected side when compared to responders [permutation test: TN site non-responders vs. responders, 5 Hz/316–438 ms; *t*(13) = 4, *p* = 0.05].

TFA in the responder group revealed a significant power in low-frequency range at the control site prior to surgery [*t*-test against baseline: 5 Hz/242–325 ms, *t*(9) = 9, *p* = 0.01; Figure 2]. At the TN site, oscillatory responses were significantly attenuated [permutation test: control vs. TN site: 4 Hz/105–176 ms, *t*(9) = 3, *p* = 0.05, effect size: *d* = 1.342; Figure 3].

In contrast, the non-responder group showed enhanced oscillatory activity at the TN site [*t*-test against baseline: 7 Hz/280–352 ms, *t*(4) = 23, *p* = 0.01; Figure 2], exceeding that at the control site [6 Hz/126–224 ms, *t*(4) = 9, *p* = 0.01]. These responses differed significantly between dermatomes [permutation test: TN vs. control site: 5 Hz/31–114 ms, *t*(4) = 2, *p* = 0.05, *d* = 1.265; Figure 3].

Notably, oscillatory patterns at the TN site significantly differed between responders and non-responders [permutation test: 5 Hz/316–438 ms, *t*(13) = 4, *p* = 0.05, *d* = 2.191; Figure 3].

## 4 Discussion

Consistent with previous studies, we observed comparable latencies of the grand-average source waveforms in contralateral S1 in TN patients (51). In contrast to previous studies reporting altered latencies or amplitudes of nociceptive-evoked potentials in TN, we did not observe significant differences between the symptomatic and control sites, or between responders and non-responders. Several factors may account for this discrepancy. All participants remained on their regular analgesic and antiepileptic medication during MEG recordings. Agents such as carbamazepine are known to affect synaptic transmission and affect peripheral nerve conduction (52) and somatosensory-evoked potentials (53). Furthermore, the relatively small sample size and interindividual variability in pain chronicity and cortical plasticity may have masked subtle effects.

In recent years, research has increasingly shifted its focus towards oscillatory activity (54, 55), as transient phase-locked responses provide limited correlation with subjective pain perception and are susceptible to habituation effects (56).

Analyses of induced low-frequency oscillations in S1 provide new insights into potential pathomechanisms of TN. The observed alterations in laser-induced low-frequency activity suggest a relevant functional pathology involving Aδ fibers and support the concept of small fiber dysfunction in classic TN (51). In line with previous studies on acute experimental pain in healthy participants, we detected oscillatory activity below 10 Hz originating from contralateral S1 at the unaffected control site (57). Interestingly, this response was attenuated at the symptomatic TN site in responders. These findings correspond to structural MRI studies reporting gray matter volume reductions in S1 in TN patients compared to healthy controls (35). Similar structural and anatomical changes in S1 have been demonstrated in other neuropathic pain conditions, such as diabetic peripheral neuropathy (58) or nociplastic pain disorders, such as complex regional pain syndrome (CRPS) (59).

Neuroplasticity describes the capacity of the nervous system to undergo functional and structural adaptations in response to internal or external stimuli, a fundamental mechanism underlying processes such as learning and memory formation. In the context of pain processing, maladaptive changes can alter synaptic efficacy, neuronal connectivity and the excitability of pain-related circuits, thereby contributing to the amplification and chronification of nociceptive signals (60, 61). Animal studies have demonstrated that chronic pain states can lead to hyperexcitability and intracortical remodeling within S1, particularly among layer 2/3 excitatory neurons, which project to other pain-relevant areas such as ACC (62).

Repetitive pain episodes seem to drive abnormal cortical recruitment and functional plasticity within somatosensory networks (63). Central facilitation and overactivation of the trigeminal nociceptive system have been observed in chronic migraine (64), chronic tension-type headache (63) and TN with concomitant facial pain (51). Elevated theta activity has been reported across various chronic pain conditions (65–67). In the present study, it remains unclear whether the increased low-frequency oscillations observed in non-responders reflect maladaptive plastic changes secondary to repeated pain episodes and trigeminal nerve injury, or whether they indicate pre-existing central susceptibilities that confer vulnerability to the development or persistence of TN.

Comparable alterations have also been identified in the time domain. Obermann et al. (51) found that TN patients with continuous pain exhibited shorter latencies and higher amplitudes of nociceptive-evoked potentials compared to those without continuous pain. The authors attributed these findings to central facilitation and sensitization (68), a mechanism characterized by heightened responsiveness of pain-related brain regions to normal or even subthreshold afferent input (69). Once established, central sensitization may become independent of peripheral input, which has important therapeutic implications: treatments targeting peripheral mechanisms might then no longer be effective.

The opposing low-frequency oscillatory patterns observed in responders and non-responders may reflect distinct underlying mechanisms of pain chronification and cortical processing. In responders, the attenuation of oscillatory activity at the symptomatic site may indicate a functional suppression or disconnection of cortical nociceptive representation due to long-standing peripheral input disruption, possibly reversible by MVD. In contrast, non-responders exhibited enhanced low-frequency activity, which may suggest maladaptive cortical hyperexcitability or central amplification that persists independently of peripheral compression. This heightened cortical synchrony could represent a marker of entrenched central sensitization or altered thalamocortical dynamics, potentially rendering purely peripheral interventions insufficient. Thus, the divergent oscillatory signatures may not only reflect the current pain state but also hint at different dominant mechanisms, peripheral vs. central, in individual patients.

These patterns could thus inform preoperative risk stratification by identifying patients with predominantly central dysfunction who may be less likely to benefit from decompression surgery alone. In such cases, neuromodulatory approaches, such as transcranial magnetic stimulation (TMS), transcranial direct current stimulation (tDCS) or invasive options like motor cortex stimulation, might serve as adjunctive or alternative strategies. Particularly in patients with elevated low-frequency activity or signs of cortical hyperexcitability, these techniques may help to normalize dysfunctional network activity and improve clinical outcomes. Ultimately, integrating oscillatory biomarkers into clinical workflows could support mechanism-based treatment decisions and pave the way for personalized therapeutic algorithms in TN.

As this study was exploratory in nature, no formal power calculation was conducted. While the limited sample size restricts the generalizability and statistical power of the findings, the observed group-level differences in cortical oscillatory dynamics provide a valuable foundation for future studies aiming to validate these effects in larger patient populations. While our findings offer valuable insights into the functional alterations associated with TN, the limited sample size constitutes a notable constraint. Future studies with larger cohorts are warranted to assess the robustness and predictive value of these results as potential diagnostic markers. Comprehensive investigations will be critical to deepen our understanding of the complex pathophysiology of TN and to inform the development of more effective diagnostic and therapeutic strategies.

In addition to the limited sample size, several other factors may have influenced our findings. The patient cohort was heterogeneous with respect to disease duration, affected trigeminal branches and medication, which may have contributed to interindividual variability in cortical responses. Although all patients met strict diagnostic criteria for classical TN, subtle differences in pain chronification, central involvement or compensatory plasticity cannot be excluded. Furthermore, all participants remained on their prescribed medication during MEG recordings. While this approach ensured ecological validity and avoided symptom destabilization, the use of antiepileptic and analgesic agents, particularly sodium channel blockers, may have affected neural excitability and oscillatory activity. Although such effects were likely present in both responders and non-responders, they represent a potential confounder and should be considered when interpreting the results.

## 5 Conclusions

MEG revealed distinct low-frequency oscillatory signatures in contralateral S1 that differentiated patients who achieved complete pain remission after MVD from those with persistent attacks. While responders showed attenuated oscillatory responses at the symptomatic site, non-responders exhibited increased activity, suggesting divergent underlying mechanisms. These findings indicate that cortical oscillations may reflect the functional state of the trigeminal nociceptive system and offer additional value beyond structural imaging for preoperative evaluation. MEG-derived measures could contribute to functional risk stratification, helping identify patients more likely to benefit from decompression and prompting consideration of adjunctive neuromodulatory strategies when central sensitization is suspected. Confirmation in larger, prospectively characterized cohorts is required to establish robustness, define clinically actionable thresholds, and standardize acquisition and analysis pipelines. If validated, integrating MEG with high-resolution imaging and clinical assessment could enable more mechanism-informed, patient-specific decision-making in TN.

## Data Availability

The raw data supporting the conclusions of this article will be made available by the authors, without undue reservation.
