# Supplementary material for: Primary somatosensory cortex oscillations in trigeminal neuralgia: laser-evoked signatures and their potential relevance to microvascular decompression
Source: Front Pain Res (Lausanne). 2025 Sep 29;6:1652354. doi: 10.3389/fpain.2025.1652354 (PMC12515803; doi:10.3389/fpain.2025.1652354)
Supplement: Supplementary file 1 [file Datasheet1.pdf]

Patients	Healthy site			Pain site		
	x	y	z	x	y	z
**VP1**	50.3	-22.4	38.5	-54.8	-7.8	35.9
**VP2**	49.4	-23.2	40.3	-54.5	-16.0	32.5
**VP3**	43.3	-11.0	33.2	-55.3	-19.9	33.5
**VP4**	45.5	-22.6	34.3	-45.1	-21.7	34.2
**VP5**	34.0	-15.8	44.6	-53.4	-18.1	34.1
**VP6**	37.3	-16.7	37.2	-45.7	-27.4	35.1
**VP7**	48.3	-15.6	30.6	-45.6	-21.5	31.7
**VP8**	-62.6	-17.2	38.1	36.1	-16.4	41.6
**VP9**	-49.6	-23.0	39.3	57.5	-13.7	43.6
**VP10**	47.6	-20.3	35.0	-38.8	-13.0	33.3
**VP11**	-52.5	-9.4	29.7	42.9	-9.3	33.1
**VP12**	-48.9	-21.9	34.9	34.2	-15.4	37.7
**VP13**	37.4	-12.9	32.7	-37.8	-19.5	34.4
**VP14**	40.3	-26.5	41.2	-38.7	-28.7	36.3
**VP15**	-52.7	-22.6	43.9	52.0	-17.4	35.9

Table 2. MNI coordinates of the contralateral primary somatosensory cortex (S1) source for the healthy and pain-affected sites in individual patients with trigeminal neuralgia. Coordinates (x, y, z) are reported in Montreal Neurological Institute (MNI) space, as determined by source localization of laser-evoked responses.
