# Supplementary material for: Primary somatosensory cortex oscillations in trigeminal neuralgia: laser-evoked signatures and their potential relevance to microvascular decompression
Source: Front Pain Res (Lausanne). 2025 Sep 29;6:1652354. doi: 10.3389/fpain.2025.1652354 (PMC12515803; doi:10.3389/fpain.2025.1652354)
Supplement: Supplementary file 2 [file Datasheet2.pdf]

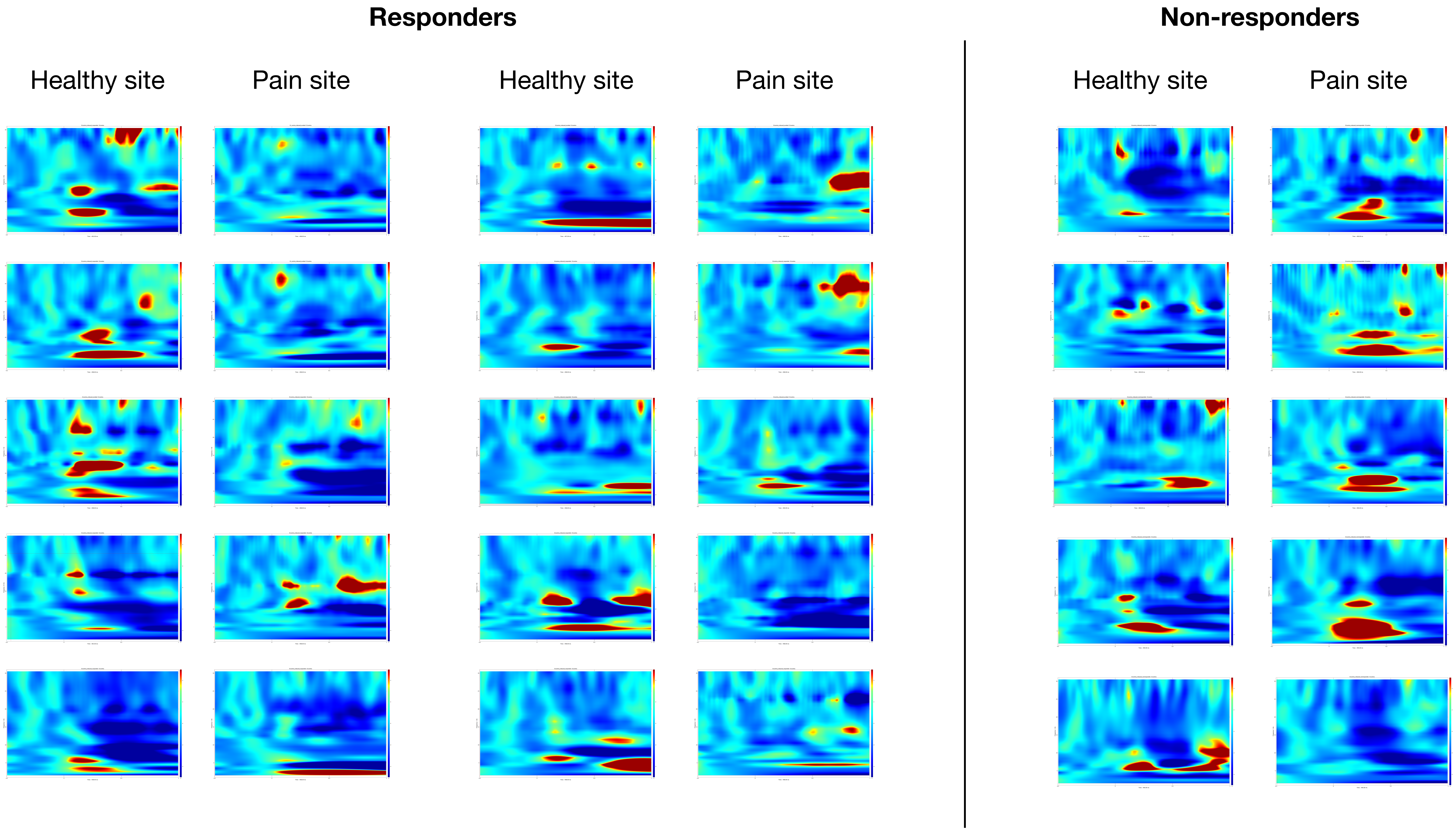
Responders
Non-responders
Healthy site
Pain site
Healthy site
Pain site
Healthy site
Pain site
